# Adolescent with 10° to 20° Cobb scoliosis during growth: efficacy of conservative treatments. A prospective controlled cohort observational study

**DOI:** 10.1186/1748-7161-7-S1-O50

**Published:** 2012-01-27

**Authors:** M Romano, A Negrini, S Parzini, S Donzelli, F Zaina, S Negrini

**Affiliations:** 1 ISICO, Milan, Italy

## Purpose/background

Usually scoliosis between 10° and 20° are not treated: in some Centres conservative preventive treatment is provided [1][2]. Aim of this study is to compare results of different type of treatments.

## Materials and methods

Population: 288 consecutive scoliosis patients over 10 years of age, curves range 10-20°, Risser 0-3 (190 Females, Age 12.8±1.5). We had 5 groups:

· Brace (BG, 40 patients): bracing 18 hours per day

· SEAS (101 patients): specific SEAS exercises (at least 3 controls per year)

· Usual Physiotherapy (UP, 70 patients): different type of exercises

· Not Compliant (NC, 46 patients): SEAS exercises 2 (or less) controls per year

· Controls (CG, 31 patients): no treatment.

Main outcome (after 12±4 months): Relative Risk of failure of treatment (worsening of 5°C or brace prescription).

## Results

At baseline BG differed from the other groups for almost all parameters. In BG failures were 10%, improvements 45%; in SEAS 16% and 30% respectively.

When compared to SEAS (and not considering BG), Relative Risk of failure was statistically significantly increased in CG (1.9, IC95 1.28-2.53) and NC (2.02, IC95 1.34-2.70), but not in UP (1.52, IC95 0.91-2.13). All patients other than SEAS had an increased Relative Risk of failure (1.74, IC95 1.22-2.26).

In BG and SEAS Trace and Cobb degrees statistically decreased (in BG also ATR), while in NC and CG humps progressed. Results were statistically better for SEAS and BG than the other groups for Trace and hump.

## Conclusions

Conservative treatment with Brace or SEAS consistently reduce the risk of progression.
